# A Case of Aplastic Anemia and Colon Cancer With Underlying Spliceosome Mutation: Is It an Incidental Finding or a Novel Association?

**DOI:** 10.7759/cureus.22632

**Published:** 2022-02-26

**Authors:** Aswani Thurlapati, Kyle Boudreaux, Srinandan Guntupalli, Richard P Mansour, Shahzeem Bhayani

**Affiliations:** 1 Internal Medicine, Louisiana State University (LSU) Health, Shreveport, USA; 2 Hematology and Oncology, Louisiana State University (LSU) Health, Shreveport, USA

**Keywords:** alternative splicing, spliceosome mutation, zrsr2 mutation, colon cancer, aplastic anemia

## Abstract

Alternative splicing is an epigenetic mechanism that plays a role in the development and function of antigen-specific lymphocytes. One such is the zinc-finger-RNA-binding-motif-and-serine/arginine-rich-2 (ZRSR2), which is clinically implicated in myelodysplastic syndrome and leukemia. Here, we present a case of a young male with myriad autoimmune conditions and adenocarcinoma of the colon in the setting of ZRSR2 mutation.

A 28-year-old male with common variable immunodeficiency disease, atopic dermatitis, autoimmune gastroenteropathy, inflammatory polyarthropathy, primary bone marrow failure, colon cancer, and family history of Lynch syndrome was admitted to our hospital for an acute flare of autoimmune enteropathy secondary to subtherapeutic tacrolimus levels.

He initially developed pancytopenia at the age of 26 years. Workup for HIV, hepatitis, cytomegalovirus, human-herpesvirus 6, parvovirus was negative. Partial thromboplastin time (PTT), international normalized ratio (INR), d-dimer, ferritin, iron profile, antinuclear antibodies (ANA) screen was unremarkable. Direct, indirect, and super-combs antibodies were undetectable. Chromosomal study for Fanconi-related chromosomal breakage and telomerase gene panel was negative. Flow cytometry did not reveal an abnormal clone. Bone marrow biopsy showed markedly hypocellular marrow with reduced trilineage hematopoiesis and 1% blasts with normal cytogenetics, immunohistochemistry, fluorescence in situ hybridization (FISH), and negative for myelodysplastic syndrome and paroxysmal nocturnal hemoglobinuria (PNH). Cincinnati inherited children’s bone marrow transplant (BMT) panel was negative. He was diagnosed with aplastic anemia and was treated with antithymocyte globulin, cyclosporine, prednisone, and currently tacrolimus. At the age of 26 years, he was diagnosed with colon cancer. Immunohistochemistry was positive for MLH1, but the confirmatory genetic testing for Lynch syndrome was negative. He underwent total proctocolectomy and ileostomy and is currently in remission. Next-generation sequencing of bone marrow revealed a germline homozygous ZRSR2 mutation.

ZRSR2 spliceosome mutations are more common in males as it’s an X-linked gene. They are seen in myelodysplastic syndrome, leukemia, increased autoimmune phenomenon, and 35 cases of colon cancer associated with this mutation are reported. In the setting of aplastic anemia and lynch negative colon cancer, we suspect our patient could have aplastic anemia due to an autoimmune phenomenon, underlying common variable immunodeficiency disease (CVID), or the new ZRSR2 mutation could be playing a role. Further studies and research is warranted to determine its true association with the disease entities. The underlying contributing factor is ZRSR2 mutation.

## Introduction

Alternative splicing is an epigenetic mechanism used by the human immune system in the development and function of antigen-specific lymphocytes [1]. One of which is the zinc-finger-RNA-binding-motif-and-serine/arginine-rich-2 (ZRSR2), which is clinically implicated in myelodysplastic syndrome and leukemia [2]. Here, we present a young male with primary bone marrow failure, adenocarcinoma of the colon, and numerous autoimmune disorders in the setting of ZRSR2 mutation.

## Case presentation

We present a chronological medical history of a 28-year-old Caucasian male with a past medical history of common variable immunodeficiency disease (CVID) with pancytopenia and colon cancer. At the age of 24 years, he was first diagnosed with auto-immune enteropathy and since then has been treated with tacrolimus. A year later, the patient was noted to have a 40 pounds weight loss with labs significant for pancytopenia, requiring numerous blood transfusions. Diagnostic workup for pancytopenia revealed the following as mentioned in Table 1. 

**Table 1:** Diagnostic workup for pancytopenia WBC: white blood cell; PT: partial thromboplastin; ANA: antinuclear antibodies; INR: international normalized ratio

Labs	Outcomes
WBC	2.5K
Hemoglobin	5.2 mg/dl
Platelets	102K
HIV, viral hepatitis, cytomegalovirus, human herpesvirus 6 parvovirus	Negative
PT/INR	11/1.14
Fibrinogen	336 mg/dl
D-dimer	353 ng/ml
Iron profile	Within normal limits
ANA screen	Negative
Direct antiglobulin test, indirect antiglobulin test, super-combs antibodies	Negative
Fanconi-related chromosomal breakage and Telomerase gene panel	Negative
Peripheral blood flow cytometry	Unremarkable
Bone marrow biopsy (BMB)	<5% hypocellular marrow with reduced trilineage hematopoiesis and 1% blasts
Bone marrow cytogenetics, immunohistochemistry, fluorescence in situ hybridization (FISH) testing	Negative
Testing for paroxysmal nocturnal hemoglobinuria and myelodysplastic syndrome	Negative
Cincinnati children’s inherited BMT panel	Negative

He was diagnosed with aplastic anemia and treated with antithymocyte globulin, cyclosporine, and prednisone. After developing cyclosporine-induced nephropathy, he was initiated on tacrolimus instead. Due to human leukocyte antigen (HLA) unmatch of his brother for allo-transplant, the patient is awaiting a matched unrelated donor for stem-cell transplantation. At the same time, as part of the anemia work, the patient has undergone endoscopic gastroduodenoscopy (EGD) and colonoscopy and was diagnosed with a stage two adenocarcinoma of the colon at the splenic flexure. Analysis for high-risk features revealed negative deficient mismatch repair (dMMR) and high levels of microsatellite instability (MSI-H). Despite a family history of Lynch syndrome, the immunohistochemistry of cancer revealed MLH1 deficiency, but no confirmatory MLH1 mutation was noted on genetic testing. BRAF mutation was also negative. He underwent total proctocolectomy and ileostomy, without any radiation or chemotherapy and is currently in remission. 

Two years later, despite being on treatment and having adequate tacrolimus levels, he was found to have worsening pancytopenia. A repeat bone marrow biopsy was performed. It revealed a 5% hypocellular marrow, 1% blasts, reduced trilineage hematopoiesis, normal fluorescence in situ hybridization (FISH) for myelodysplastic syndrome (MDS), and 46 XY,del(13)(q12q22)/46,XY. Next-generation sequencing of bone marrow was performed and the patient was found to have a germline hemizygous ZRSR2 c.1147 C>G 100% variant allelic frequency (VAF) mutation. 

## Discussion

Pancytopenia is a common entity found in clinical practice. A comprehensive workup is necessary to determine the underlying etiology. After ruling out drugs, infections, malignancy, and congenital causes, our patient was diagnosed with aplastic anemia with trilineage hypocellular bone marrow. Studies suggest, one of the pathophysiologic causes of aplastic anemia includes autoantibody-mediated pancytopenia due to the result of impaired B-cell maturation. This particularly is seen in patients with underlying immunologic disorders such as CVID and other autoimmune disorders. Studies also suggest that it can also be a sequela of polygenetic or epigenetic defects in the hematopoietic stem cells or the immune system leading to autoimmune reactions [3]. Genetic mapping using next-generation sequencing (NGS) enhances current and identifies novel pathophysiology, prediction of therapeutic benefits of existing agents, and developing novel targeted therapy. It also provides a new link between aplastic anemia and its clonal complications, such as evolution into MDS and leukemia [4]. Novel discoveries of molecular genetics in aplastic anemia led to a paradigm shift in pathophysiology from solely an autoimmune disorder to a multifactorial mechanism consisting of cytogenetic abnormalities, recurrent somatic mutations, germline mutations, telomere attrition, and immune dysregulation [5]. One such epigenetic defect includes ZRSR2 mutation, which encodes for U2 small nuclear ribonucleoprotein auxiliary factor 35 kDa subunit-related protein 2 that takes part in RNA splicing. 

Spliceosome mutations, such as ZRSR2 mutation are rare epigenetic mechanisms that have been associated with hematologic malignancy. Mutations of ZRSR2 are more common in males as it’s an X-linked gene. They are reported in 4.3% of myelodysplastic syndrome, 1.5% of acute myeloid leukemia, chronic myelomonocytic leukemia, and chronic lymphocytic leukemia. Studies suggest the majority of the mutations reported are somatic heterozygous missense mutations [6-8]. Here, we report a patient with underlying homozygous germline mutation of ZRSR2 in the setting of aplastic anemia. However, with an underlying CVID, our patient’s development of aplastic anemia could be multifactorial and not just solely because of the underlying mutation. Also, more research is warranted to determine if an underlying spliceosome mutation in aplastic anemia puts them at a higher risk for long-term complications such as the development of MDS and leukemia. 

Compared to hematologic diseases, very few spliceosome alterations are noted in solid tumors. According to the American Association of Cancer Research (AACR) Genomics Evidence Neoplasia Information Exchange (GENIE) Consortium, around 35 cases of colon cancer associated with the ZRSR2 mutation are reported [9]. Our patient was diagnosed with adenocarcinoma of the colon, negative for Lynch syndrome despite strong family history. However, CVID and autoimmune enteropathy are also known to be pro-oncogenic leading to the development of cancer. Hence, ZRSR2 mutation may be a co-incidental finding in our patient or could possibly have played a substantial role in the development of both aplastic anemia and colon cancer. Hence, we believe further reports with novel associations and further research is warranted to determine new pathophysiologic mechanisms and attribute novel associations. 

In addition to pathophysiology, determining novel genetic targetable associations in diseases can provide a novel approach to treatment. One example includes the use of splicing modulator compounds, such as Sudemycin which binds to SF3B1 protein inducing a conformational change to modulate the pre-mRNA splicing [10]. Studies suggest that cells expressing mutant spliceosome genes have increased sensitivity to pharmacological agents. Seiler et al. proved the potential use of a novel in-trial compound called H3B-8800, which is an oral small-molecule splicing modulator in spliceosome-mutant cancers [11]. Cretu et al. reviewed various SF3B complex binding compounds, like pladienolide B, herboxidiene (GEX1A), and spliceostatin A (SSA) that induce changes in alternative splicing patterns [12]. The SF3B1 arrests spliceosome assembly, making the base-pairing interaction between U2 and the intron altered [13]. Since ZSZR encodes for U2, this potentially shows how these compounds could be used under trials for diseases with underlying ZRSR2 mutations. According to My Cancer Genome, currently, two clinical trials about ZRSR2 mutations are open.

## Conclusions

Our patient is unique as he has a combination of hematologic and oncologic disorders with an underlying ZRSR2 spliceosome mutation. However, it should still be taken as a caution with this novel association in our patient. With an underlying history of CVID which is also prone to developing aplastic anemia through autoimmune mechanisms, one can only speculate the probable underlying mechanisms. Hence, the ZRSR2 mutation might be an incidental finding or might have played a pivotal role in our patient. Hence, we believe with the advent of NGS, understanding the role of genetic landscape in primary bone marrow failure and cancer will help determine novel associations. Further research is warranted to determine if ZRSR2 mutation is an incidental finding or a pivotal novel discovery.
